# Integrating Prior Knowledge Using Transformer for Gene Regulatory Network Inference

**DOI:** 10.1002/advs.202409990

**Published:** 2024-11-28

**Authors:** Guangzheng Weng, Patrick Martin, Hyobin Kim, Kyoung Jae Won

**Affiliations:** ^1^ Biotech Research and Innovation Centre (BRIC) University of Copenhagen Ole Maaløes Vej 5 Copenhagen 2200 Denmark; ^2^ Department of Computational Biomedicine Cedars‐Sinai Medical Center Los Angeles CA 90069 USA

**Keywords:** deep learning, gene regulatory networks, inference, large language model, temporal convolutional network, transformer

## Abstract

Gene regulatory network (GRN) inference, a process of reconstructing gene regulatory rules from experimental data, has the potential to discover new regulatory rules. However, existing methods often struggle to generalize across diverse cell types and account for unseen regulators. Here, this work presents GRNPT, a novel Transformer‐based framework that integrates large language model (LLM) embeddings from publicly accessible biological data and a temporal convolutional network (TCN) autoencoder to capture regulatory patterns from single‐cell RNA sequencing (scRNA‐seq) trajectories. GRNPT significantly outperforms both supervised and unsupervised methods in inferring GRNs, particularly when training data is limited. Notably, GRNPT exhibits exceptional generalizability, accurately predicting regulatory relationships in previously unseen cell types and even regulators. By combining LLMs ability to distillate biological knowledge from text and deep learning methodologies capturing complex patterns in gene expression data, GRNPT overcomes the limitations of traditional GRN inference methods and enables more accurate and comprehensive understanding of gene regulatory dynamics.

## Graphical abstract

GRNPT is a powerful tool for reconstructing gene regulatory networks (GRNs) from single‐cell data. By combining gene embeddings from large language models with temporal information, GRNPT accurately identifies regulatory relationships between genes. Its ability to generalize to unseen data makes it highly adaptable. Even with limited prior knowledge of gene regulations, GRNPT can effectively reconstruct large and reliable GRNs.

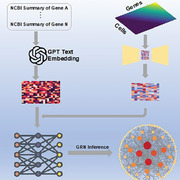

## 1 Introduction

Gene regulatory networks (GRNs) represent complex interactions between genes and their regulatory elements. Despite their importance in understanding biological processes, obtaining GRNs remains a significant challenge for experimental approaches. As a result, computational approaches have been extensively studied to infer GRNs from available data.

Traditionally, time‐series bulk transcriptomic data have been used to infer GRNs based on the underlying assumption that co‐regulated genes exhibit correlated expression patterns over time.^[^ ^1^ ^]^ Later, single‐cell RNA sequencing (scRNA‐seq) data have been widely used as they can provide co‐expression information at the individual cell level.^[^ ^2^ ^]^ Unsupervised learning methods have been popular in GRN inference due to the scarcity of large‐scale, high‐quality training datasets. Several unsupervised computational methods have been developed to infer GRNs from gene expression data. For instance, GENIE3 utilizes random forests for feature importance ranking.^[^ ^3^ ^]^ For each target gene, the random forest algorithm predicts its expression level based on input gene expression data.^[^ ^3^ ^]^ Through multiple training iterations of different decision trees, GENIE3 evaluates the contribution of each input gene to the target gene. SCODE employed ordinary differential equations (ODEs) to detect the relationships among genes from scRNA‐seq trajectories.^[^ ^4^ ^]^ Mutual information has been applied for PIDC^[^ ^5^ ^]^ to identify statistically dependent genes, potentially reflecting regulatory relationships. TENET^[^ ^6^ ^]^ and SCRIBE^[^ ^7^ ^]^ leverage transfer entropy (TE) to identify potential causal relationships. SINCERITIES employs regularized linear regression to assess dependencies between gene expression levels and identify potential regulatory relationships.^[^ ^8^ ^]^ Unsupervised methods typically require significant computational resources and lack the ability to generalize to new datasets.

The growing availability of extensive datasets has facilitated the development of supervised learning algorithms for reconstructing GRNs.^[^ ^8^, ^9^, ^10^ ^]^ These methods leverage public resources and deep learning to achieve faster and more accurate inference. Often, chromatin immunoprecipitation followed by sequencing (ChIP‐seq) data has been suggested as the training set.^[^ ^11^ ^]^ DGRNS combines the strengths of recurrent neural networks (RNNs) and convolutional neural networks (CNNs) to process time‐dependent and spatially correlated information, thereby enhancing the ability to distinguish related gene pairs from unrelated ones.^[^ ^10^ ^]^ GMFGRN integrated matrix factorization and graph neural networks (GNNs) to learn representative gene embeddings and determine regulatory relationships.^[^ ^12^ ^]^ DeepRIG is a semi‐supervised deep learning framework that utilizes a graph autoencoder (GAE) to decode complex gene regulatory relationships.^[^ ^9^ ^]^ For this, DeepRIG calculates correlation coefficients between each pair of genes to construct a correlation‐based co‐expression network and converts it into a prior regulatory graph.

Despite outperforming unsupervised algorithms, supervised approaches still have several limitations. First, while public databases contain a wealth of information on gene function and protein interactions, current supervised deep learning methods often fail to effectively integrate this knowledge, limiting their ability to fully exploit available information. Second, deep learning models trained on data from specific cell types may not generalize well to other conditions or cell types. Third, deep learning methods often require large datasets for effective training, which can be a bottleneck, especially for studying rare cell types or under‐represented biological processes with limited data availability.

To overcome limitations of traditional supervised learning in GRN inference, we developed GRNPT. GRNPT is a novel Transformer‐based method that combines two components, one incorporates general biological knowledge and the other learns domain specific rules from scRNA‐seq data. To incorporate general biological knowledge, GRNPT uses the embeddings from large language models (LLMs). Specifically, GRNPT employs GenePT,^[^ ^13^ ^]^ which is trained on the contents from NCBI database^[^ ^14^ ^]^ using GPT‐3.5 embedding model.^[^ ^15^ ^]^ To provide domain specific rules, GRNPT uses a TCN autoencoder^[^ ^16^ ^]^ trained on the scRNA‐seq data aligned based on the cell trajectory. The trained TCN captures temporal dynamics in gene expression. These are integrated to an advanced Transformer model.^[^ ^17^ ^]^ A Transformer is trained using known regulatory knowledge (e.g., ChIP‐seq). The Transformer learns latent patterns of gene regulatory pairs and reconstructs the GRN through a decoder. This architecture enables GRNPT to significantly reduce training data requirements and generalize its predictions to unseen data.

We rigorously evaluated GRNPT's effectiveness using simulated data from the Beeline framework.^[^ ^18^ ^]^ GRNPT substantially outperformed other supervised methods, even when trained only on 10% of the data. In contrast, competing approaches typically require more than half the data to achieve similar performance. More importantly, GRNPT exhibits strong generalizability. A model trained on one cell type can be applied to others with limited compromise in the performance. In addition, GRNPT trained on known regulators can predict the potential regulatory relationships of unseen regulators. This level of generalizability surpasses existing supervised and unsupervised methods for GRN prediction.

## 2 Results

### 2.1 GRNPT is a Generalizable GRN Inference Tool that Integrates Public Knowledge and Gene Co‐Regulation Patterns

**Figure 1:**
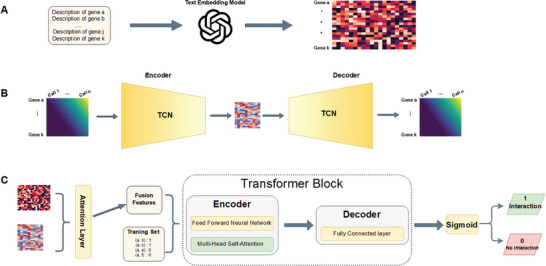
Overview of GRNPT Framework A) GenePT generates gene embedding vectors, each with 1536 numerical dimensions. Trained on textual gene descriptions from the NCBI database, these vectors capture essential biological information for accurate GRN inference. B) A TCN autoencoder extracts latent features capturing temporal dependencies among genes from scRNA‐seq data aligned based on the trajectory. C) An attention layer integrates LLM and TCN features to assign weights to gene features. A Transformer model, using multi‐head attention and feed‐forward networks, predicts gene regulatory interactions based on these weighted features. The model is trained on ChIP‐seq data with negative sampling.

GRNPT prioritizes leveraging public resources to achieve generalizability in GRN inference. To capitalize on publicly available information about genes, their functions, and regulatory relationships, GRNPT incorporated gene embedding vectors generated by GenePT. GenePT utilizes text descriptions of individual genes from the NCBI database. These text descriptions are processed through a GPT‐3.5 embedding model, allowing to capture biological information for various tasks, including gene clustering, interaction prediction, and protein network analysis.^[^ ^13^ ^]^ GRNPT directly utilized the 1536‐dimensional numerical vector generated by GPT‐3.5 embedding model for each gene (**Figure** **1**A). These vectors encapsulate biological information, enabling GRNPT to infer GRNs with generalization capabilities.

To capture regulatory relationships between genes, we arrange the expression levels along the single cell trajectory (Figure 1B). This ordered data are fed into a TCN autoencoder. TCNs are a specific type of neural networks adept at handling sequential data like time series,^[^ ^19^ ^]^ making them well‐suited for capturing the temporal dependencies within the gene expression trajectories. Compared to other architectures, TCNs are more efficient at handling long sequences due to their lower computational complexity. In GRNPT, the TCN autoencoder extracts latent features that capture temporal gene co‐regulation patterns (Figure 1B).

To integrate the features extracted from the TCN autoencoder and GenePT embeddings, we incorporated an attention layer (Figure 1C). This layer concatenates the two features (i.e., TCN features and GenePT embeddings) and assigns attention scores using a linear layer followed by a softmax function. These scores represent the relative importance of each feature for the current input. By dynamically adjusting these scores during each forward pass, the attention mechanism enables GRNPT to capture the changing relationships and varying importance between the features. The weighted features and labeled edges serve as input to a network prediction model primarily based on the Transformer architecture.^[^ ^17^ ^]^ Transformers can effectively handle long‐range dependencies within the input sequences in parallel using self‐attention mechanisms.^[^ ^20^ ^]^ This capability can be useful in capturing the complex relationships between genes and their regulators. Transformers have been widely used in many areas including natural language processing (NLP), computer vision, and audio and speech processing, healthcare, and internet of things (IoT).^[^ ^21^ ^]^ During the encoding phase, the model employs multi‐head attention mechanisms^[^ ^22^ ^]^ and feed‐forward neural networks to process the features. In the decoding stage, the model extracts the features from the source and the target nodes based on the edge labels, subsequently generating predictions through fully connected layers (Figure 1C).

To train the Transformer, we used the publicly available ChIP‐seq datasets^[^ ^23^ ^]^ as positive examples. To establish a balanced dataset for each cell line, a negative regulatory pairs were randomly generated for every positive examples. This generates negative examples to balance the datasets and prevent overfitting on positive interactions. We trained the model using the Adam optimizer^[^ ^24^ ^]^ using binary cross‐entropy loss as the objective function. During training, the model makes predictions (forward propagation) and then adjusts its internal parameters (backpropagation) to minimize the loss.

### 2.2 GRNPT Outperformed other Supervised Learning Approaches Even with a Low Training Data Size

To assess GRNPT's effectiveness, we utilized six scRNA‐seq datasets from Beeline,^[^ ^18^ ^]^ hESC, mDC, mHSC‐L, mHSC‐GM, and mHSC‐E (Method). Following the preprocessing protocols from Scanpy,^[^ ^25^ ^]^ we identified 500 highly variable genes for each dataset (see Method).

For the assessment, we compared GRNPT with established unsupervised methods including GENIE3,^[^ ^3^ ^]^ GRNBOOST2,^[^ ^26^ ^]^ LEAP,^[^ ^27^ ^]^ PIDC,^[^ ^5^ ^]^ SCODE,^[^ ^4^ ^]^ and SINCERITIES^[^ ^8^ ^]^ and supervised approaches including DGRNS,^[^ ^10^ ^]^ GMFGRN,^[^ ^12^ ^]^ and DeepRIG.^[^ ^9^ ^]^ For supervised approaches, we included positive relationships obtained from ChIP‐seq databases^[^ ^23^ ^]^ (see Method).

For GRNPT, we employed a very strict tenfold cross‐validation, 10% of the data were used as the training set and the remaining 90% as the test set. In contrast, we allowed more lenient criterion for other supervised approaches as they require large amount of labeled data. In mESC, for instance, GMFGRN is trained using over 15 000 labels for specific TFs targets which is larger than 50% of the dataset. DGRNS also uses 60% of the data as the training. Once trained, the relationships between the TFs and the top 500 variable genes were predicted for each method. Based on the ChIP‐seq data, we assess the performance using the area under the precision‐recall curve (AUPRC) and the area under the receiver operating characteristic (AUROC) (Method).

**Figure 2:**
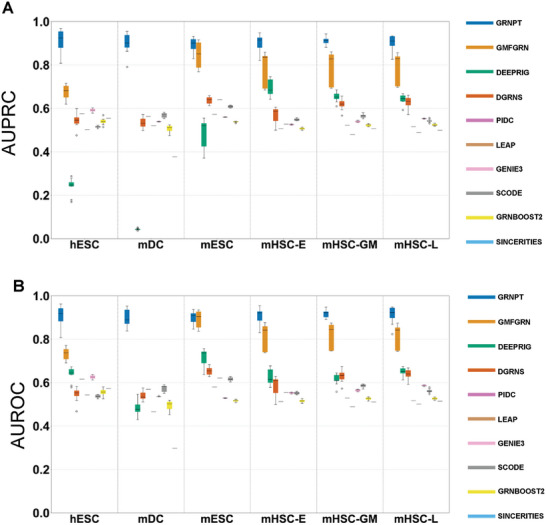
Performance comparison of predicting GRN reconstruction A) The AUPRC is compared across various datasets (hESC, mDC, mESC, mHSC‐E, mHSC‐GM, mHSC‐L). B) The AUROC is compared across various datasets. GRNPT outperformed other approaches even with 10% of the data for training. In contrast, other supervised approaches use more than 50% of the data for training.

Across all the tests we performed, GRNPT consistently outperformed other approaches (**Figure** **2** and S1, Supporting Information). The performance of GRNPT is remarkable in that it only uses 10% of the data as a training set, while other supervised approaches used more than 50% of them for training. Supervised approaches like DRGNS and GMFGRN performed better than unsupervised approaches. However, DeepRIG showed AUPRC less than 0.3 for hESC and mDC potentially due to overfitting. GMFGRN did not run on the mDC dataset.

We further included additional metrics including Accuracy (ACC), Matthews Correlation Coefficient (MCC), True Positive Rate (TPR), True Negative Rate (TNR), Error Prediction Rate (EPR), and False Discovery Rate (FDR) (Figure S2, Supporting Information). The high ACC and MCC values demonstrate its robustness and balanced predictive capabilities, while the well‐balanced TPR and TNR indicate its effectiveness in accurately identifying true positives and true negatives. GRNPT consistently demonstrated superior performance across these metrics. Furthermore, GRNPT exhibits lower EPR and FDR values, indicating a reduced rate of erroneous predictions and false positives.

To further ensure a fair comparison of GRNPT's performance, we tried to train GRNPT and other approaches using 30% of the data as a training set. For this test, we included additional three supervised GRN inference methods designed for time series scRNA‐seq data including TDL,^[^ ^28^ ^]^ dynDeepDRIM,^[^ ^29^ ^]^ and scTGRN.^[^ ^30^ ^]^ Across all metrics, GRNPT demonstrated superior performance (Figure S3, Supporting Information). GMFGRN, which showed the second‐best performance in cross‐validation, exhibited a significant decline in its performance due to its dependence on large training samples.

To determine the required amount of data to train GRNPT, we evaluated its performance while varying the portion of training data on the mESC dataset. As anticipated, with minimal training (less than 0.01% of the total data), the model's performance was comparable to random chance (AUROC and AUPRC ≈0.5) (Figure S4, Supporting Information). However, even a small amount of data (5%) significantly boosted GRNPT's performance, achieving AUROC and AUPRC of ≈0.8, surpassing other methods. Further increasing the training data to 10% led to continued improvements, although the gains became more marginal as the dataset size grew (Figure S4, Supporting Information).

### 2.3 GRNPT Infers Relationships of Untrained Datasets

Traditional GRN inference methodologies lack the ability to generalize to unseen biological contexts. Models trained on one dataset typically demonstrate limited ability in predicting gene regulatory relationships in new, unseen data. This lack of transferability is particularly problematic for supervised approaches, which often rely on substantial amounts of carefully curated data.

**Figure 3:**
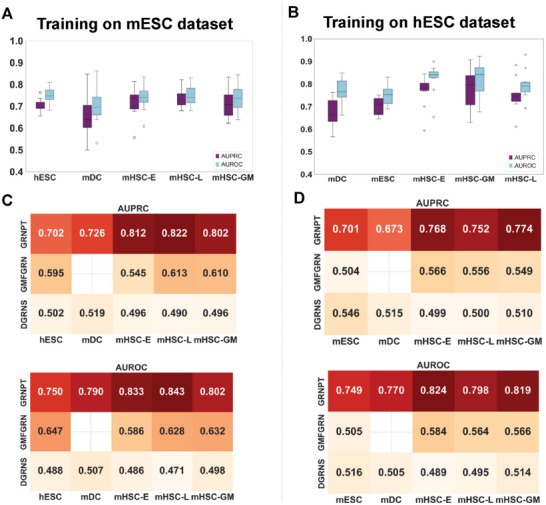
GRNPT exhibits generalizability to previously unseen cell types A) GRNPT was trained on the mESC dataset and tested on other datasets (hESC, mDC, mHSC‐E, mHSC‐L and mHSC‐GM) excluding shared regulatory pairs. The AUPRC and AUROC scores demonstrate GRNPT's ability to generalize. B) GRNPT was trained on the hESC dataset and evaluated on mESC, mDC, mHSC‐E, mHSC‐L, and mHSC‐GM datasets. C) Heatmaps comparing AUPRC and AUROC scores across GRNPT, GMFGRN, and DGRNS on hESC, mDC, mHSC‐E, mHSC‐L, and mHSC‐GM datasets. D) Heatmaps of AUPRC and AUROC scores showing the performance of GRNPT, GMFGRN, and DGRNS after training on hESC. GMFGRN does not run on the mDC dataset.

To demonstrate GRNPT's ability to generalize to unseen data, we trained a model on the mESC dataset and then applied it to entirely new datasets – hESC, mHSC‐L, mHSC‐GM, mHSC‐E, and mDC. In this evaluation, we utilized all labels for the test datasets while ensuring that only 10% of the mESC data was used for training. Additionally, we excluded any regulatory pairs shared between mESC and the test datasets to avoid overlap. Despite being trained solely on the mESC dataset, GRNPT performed remarkably well on entirely new datasets showing AUPRC and AUROC over 0.7 hESC, mDC, mHSC‐L, mHSC‐GM and mHSC‐E (**Figure** **3**A,C).

We further assessed if other approaches could predict the relationships of unseen cell types. We chose GMFGRN and DGRNS as they performed better than other approaches in the cross‐validation. In this evaluation, we followed the original authors' recommendation for GMFGRN and DGRNS, using two‐thirds of the data as the training set. In contrast, GRNPT used only 10% of the data for training. The performance of GMFGRN and DGRNS showed AUPRC < 0.62 and AUROC < 0.65 across all datasets (Figure 3C).

To further test GRNPT's ability of generalization, we conducted another test by training it on the hESC dataset and then evaluating on mESC, mDC and mHSC lines (GM, E, and L). Again, GRNPT showed the better performance than GMFGRN and DGRNS and the AUPRC and AUROC of GMFGRN and DGRNS stayed below 0.6 (Figure 3D).

For a fair comparison, we further tested the performance by allowing two‐thirds of data for training GRNPT using mESC and hESC. GRNPT showed a noticeable improvement compared to using only 10% of the data as the training set (Figure S5A, Supporting Information) even though testing with the data from unseen cell types.

We further investigated the amount of training data required for GRNPT to achieve generalizable performance. For this test, we used mESC data as training and mDC data as testing. Interestingly, performance steadily improved as the training portion of the mESC dataset increased, reaching a peak at ≈30%. However, further increases in training data resulted in fluctuations (Figure S5B, Supporting Information).

### 2.4 GRNPT Infers Relationships of Untrained Regulators

A drawback of supervised methods is their inability to generalize relationships beyond the regulators included in training data. Since these models are often trained on ChIP‐seq data, they struggle to predict relationships for regulators lacking sufficient ChIP‐seq data.

To evaluate GRNPT's ability to predict for unseen regulatory pairs, we performed an experiment focused on unseen transcription factors (TFs). We trained the model on a subset of known TFs and then tested its capacity to predict relationships for unseen TFs. This process was repeated for each dataset.

**Figure 4:**
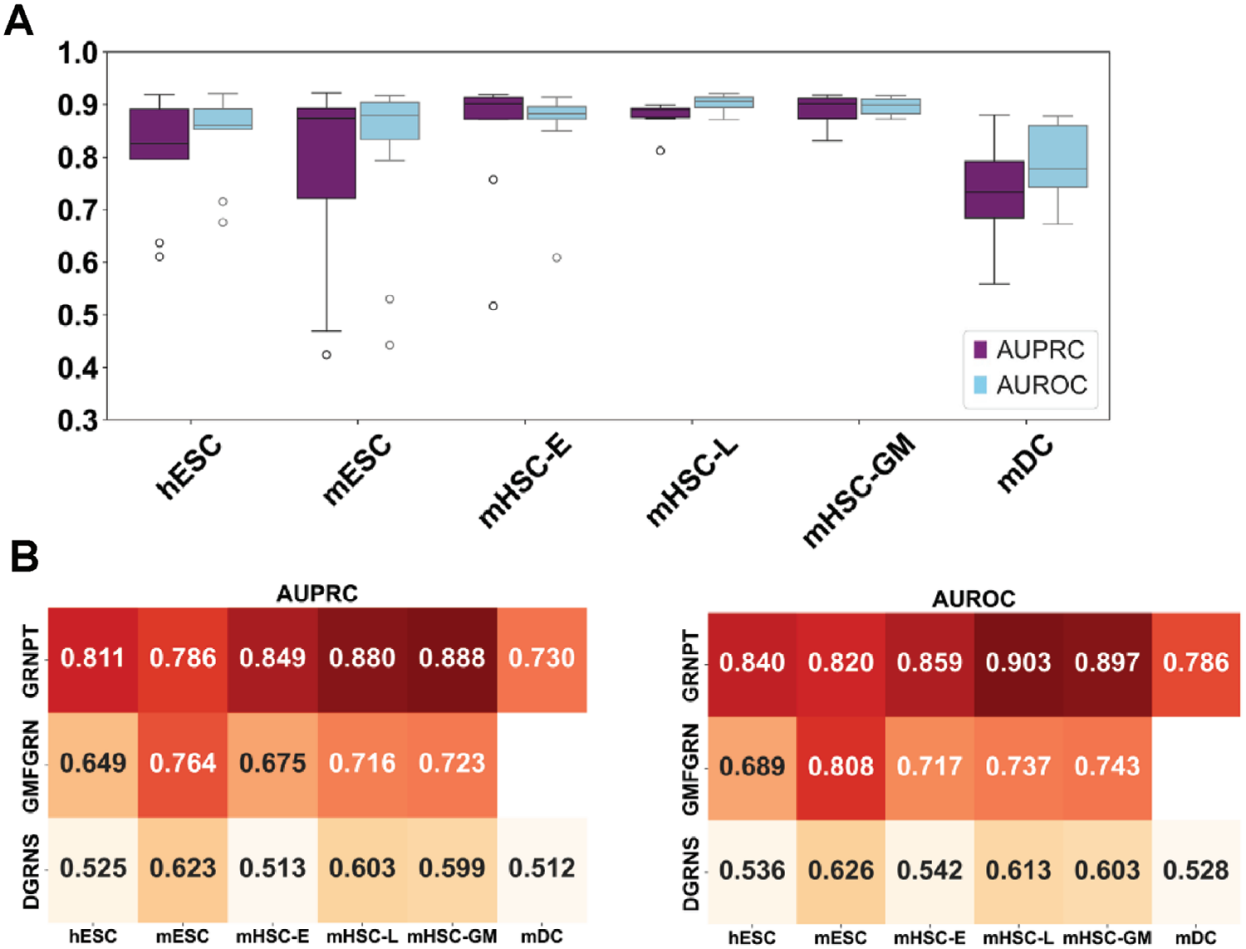
GRNPT exhibits generalizability to previously unseen regulators A) GRNPT was trained on a subset of known TFs and tested on completely unseen TFs across various datasets. It achieved high mean AUPRC and AUROC scores (>0.8) for mHSC‐E, mHSC‐L, and mHSC‐GM, and slightly lower scores for mDC due to limited ChIP‐seq data. B) Heatmaps comparing GRNPT, GMFGRN, and DGRNS on unseen TFs across the same datasets. GRNPT outperforms other methods, particularly in mHSC‐E, mHSC‐L, and mHSC‐GM, where it maintains AUPRC and AUROC scores above 0.8. GMFGRN does not run on the mDC dataset.

For these unseen regulators, we observed a remarkable performance for mHSC‐E, mHSC‐L, and mHSC‐GM datasets with the mean AUPRC and the mean AUROC > 0.8 (**Figure** **4**A). The performance is a bit lower for mDC (mean AUPRC and the mean AUROC > 0.7), mainly due to limited number of ChIP‐seq data for this cell type. These results clearly show that the performance of GRNPT is better than other supervised approaches even without training on the same TFs (Figure 4A). While GMFGRN showed some capability to predict unseen regulators, its performance was poorer than GRNPT. DGRNS performs the worst (Figure 4B). These results indicate that GRNPT can infer relationships for unseen TFs and even non‐DNA‐binding regulators.

### 2.5 GRNPT Demonstrates Excellent Run time Performance

**Figure 5:**
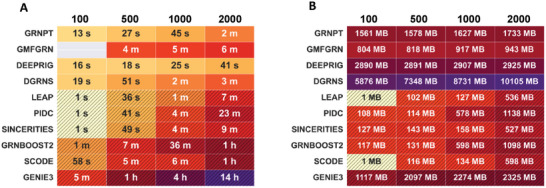
Comparison of computational complexity on mHSC‐L data with different sizes. A) Execution times for various software tools were measured on mHSC‐L datasets of varying sizes, ranging from 100 to 2000 genes. B) The memory usage of GRNPT and other methods is also shown across different data scales. The slanted grid marker in the figure indicates an unsupervised method.

To compare the running time of GRNPT against other methods, we selected mHSC‐L data with gene scales of 100, 500, 1000, and 2000 genes, which corresponds to GRN sizes of 500, 2900, 4800, and 9700 edges, respectively. For this evaluation, we excluded the time required for gene summary embedding in GRNPT and pre‐training time for other methods. GRNPT exhibited excellent performance across all gene scales. Unsupervised methods often experience a significant increase in computation time as the number of genes grows. In contrast, supervised approaches, such as GRNPT, exhibit a nearly linear increase in running time, making them more scalable for large‐scale gene expression data (**Figure** **5**A).

We also benchmarked the memory usage on datasets of varying scales, ranging from 100 to 2000 genes. Due to their lack of sample training, unsupervised methods generally have simpler model structures, resulting in significantly lower memory consumption compared to supervised methods. Among the supervised methods, GRNPT—which consists of both TCN and Transformer components—ranked second in memory usage, following GMFGRN. This is because both the TCN and Transformer models require substantial memory to load data (Figure 5B).

### 2.6 GRNPT is a Robust Approach for GRN Inference

We further investigated if GRNPT is influenced by factors such as cell number, data sparsity (dropout rate), and the number of genes using the mHSC‐E dataset. We systematically assessed GRNPT's performance under varying cell counts (100–1000), dropout rates (10–90%), and gene numbers (500–2000). Results demonstrated consistent high performance across all tested conditions, with AUPRC and AUROC values consistently above 0.8 (Figure S6, Supporting Information). Even under extreme conditions, such as 80% data dropout, GRNPT maintained excellent performance. These results demonstrate GRNPT's robustness to the common challenges encountered in scRNA‐seq data analysis.

### 2.7 GRNPT Infer Potential GRNs from the scRNA‐seq Data from Developing Mouse Pancreas

**Figure 6:**
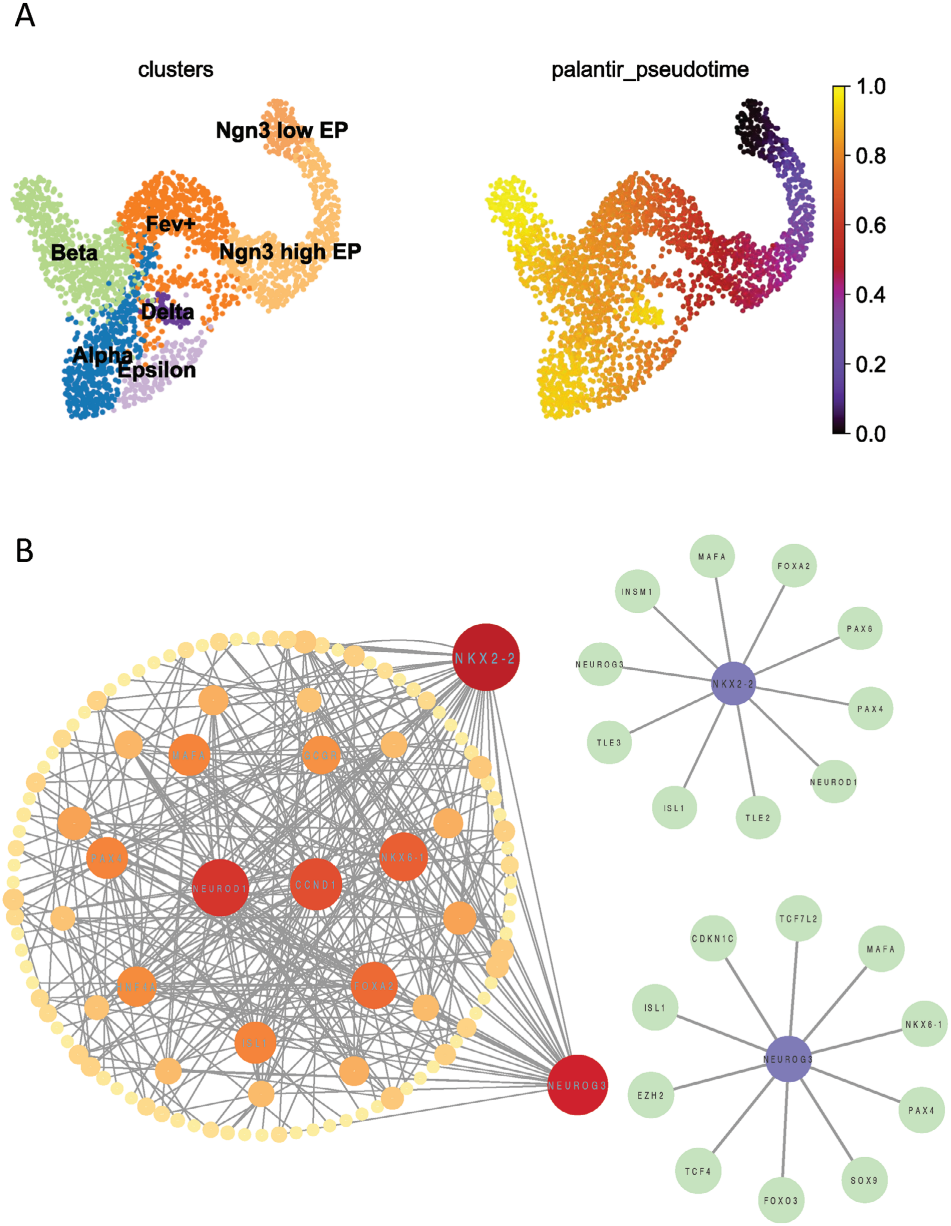
The GRN inferred from scRNA‐seq data during mouse pancreas development. A) The cell type and the trajectory identified using the scRNA‐seq from mouse pancreas at the E15.5 stage. Endocrine cells, marked by Neurog3, differentiate into α, β, δ, and ε cells. B) GRNPT inferred the regulatory network controlling pancreatic development, focusing on Neurog3 and Nkx2‐2. The top 10 gene regulatory pairs with the highest scores for NKX2‐2 and NEUROG3 are highlighted.

Next, we tested GRNPT using the scRNA‐seq data from mouse pancreas at the E15.5 stage. We directly obtained the processed mouse pancreatic scRNA‐seq data from Cell Rank^[^ ^31^ ^]^ and visualized the cell type and the trajectory in **Figure** **6**A. We utilized 500 highly variable genes, including crucial TFs such as Neurog3 and Nkx2‐2. Instead of ChIP‐seq data, we utilized 57 gene regulatory pairs from the STRING database^[^ ^32^ ^]^ as training data. These regulatory pairs include three regulatory pairs associated with Neurog3 and two with Nkx2‐2. The inferred network is shown in Figure 6B. Assessment using the interactions obtained from STRING database showed an AUPRC of 0.69 and an AUROC of 0.78 despite the database's incompleteness.

We show the top 10 gene regulatory pairs with the highest scores for both NKX2‐2 and NEUROG3. We further investigated literature associated with the identified interactions. For instance, Neurog3 upregulates Nkx2.2, promoting pancreatic α and β cell development.^[^ ^33^ ^]^ Neurog3 downregulates Sox9, a relationship reciprocally inhibited during endocrine differentiation.^[^ ^34^, ^35^ ^]^ Additionally, Ezh2 deficiency enhances Neurog3 expression by reducing H3K27me3 levels.^[^ ^36^ ^]^ Cdkn1c, a cell cycle inhibitor, exhibits a complex interplay with Neurog3, involving both reciprocal suppression and activation.^[^ ^37^ ^]^

GRNPT also identified several key regulatory relationships involving Nkx2.2, a critical factor in pancreas development.^[^ ^33^, ^38^ ^]^ For instance, Nkx2.2 directly activates Neurod1, promoting β‐cell differentiation, while Neurod1 conversely inhibits Nkx2.2.^[^ ^39^ ^]^ Additionally, Nkx2.2 upregulates Isl1, which in turn induces Nkx2.2 expression, forming a positive feedback loop essential for endocrine cell differentiation and maturation.^[^ ^40^, ^41^, ^42^ ^]^

## 3 Discussion

The field of computational biology is experiencing an explosion of data generation. This ever‐growing wealth of information, encompassing diverse types of biological data like gene expression profiles and protein‐protein interactions, plays a critical role in advancing the performance of many prediction approaches.^[^ ^13^ ^]^ For GRN inference, the ability to leverage available data will be particularly impactful.

Deep learning has been increasingly adopted in supervised approaches to infer GRNs, leveraging the abundance of available data.^[^ ^8^, ^9^, ^10^ ^]^ ChIP‐seq data, which identify TFs and their target genes, have been a common training resource for these models. However, the reliance on ChIP‐seq data limits their applicability in scenarios where such information is unavailable. While single‐cell assay for transposase‐accessible chromatin sequencing (scATAC‐seq) can be a valuable tool for generating training data,^[^ ^43^ ^]^ its direct integration does not ensure generalization. There is a pressing need for supervised methods that can effectively integrate diverse data sources and exhibit strong generalization capabilities to new datasets.

To develop a supervised GRN inference approach with generalization capability, we introduced GRNPT, a novel framework incorporating LLM and TCN. LLMs geared by GPT enabled us to use massive knowledge contained in text form. For biological context, a number of LLM based approaches were created including GenePT and scGPT.^[^ ^44^ ^]^ GRNPT employed GenePT, a model trained on the GPT‐3.5 embedding model using the NCBI database. This integration enables GRNPT to encode comprehensive knowledge about genes, their functions, and interactions. By providing a broader knowledge of gene regulation than ChIP‐seq data alone, LLMs enhance GRNPT's generalization capabilities, making it less susceptible to overfitting and more effective in predicting regulatory relationships for unseen regulators. Without LLM embeddings, GRNPT would function similarly to other supervised learning approaches with limited generalization ability.

In parallel, GRNPT employed TCN autoencoder to captures the dynamic co‐regulation of gene expressions. Intuitively, this is similar to our prior works utilizing information theory,^[^ ^6^ ^]^ where we successfully inferred regulatory interactions by analyzing fluctuations in gene expression levels.

By combining LLM embeddings and TCN embeddings, GRNPT achieved enhanced generalization capabilities and required less training data. The domain specific information is captured within the TCN, where aligned scRNA‐seq data are represented in a low‐dimensional space. After integrating domain specific information with the general knowledge obtained from the LLM embedding, GRNPT effectively identifies gene regulatory relationships through a training process.

Unlike traditional methods that require retraining for each specific dataset and set of regulators, GRNPT excels in transferring its knowledge. A model trained on an scRNA‐seq dataset from one cell type can be effectively applied to predict GRNs from a completely different cell type (Figure 3). Similarly, a model trained on a set of known regulators can be used to identify target genes for entirely new regulators (Figure 4). GRNPT's generalization capabilities are particularly impressive, as other supervised approaches often require training on matching cell types and with positive reference data (e.g., ChIP‐seq). Consequently, regulatory relationships lacking positive reference data due to insufficient binding information cannot be investigated using traditional methods. Unsupervised learning approaches have also faced challenges in applying models trained on different cell types. Even in case that there are no public resources for specific domain, GRNPT can still use TCN to predict relationships from scRNA‐seq data. The unique architecture of GRNPT suggests a promising avenue for integrating public data to enhance GRN inference.

GRNPT's generalization ability shares similarities with zero‐shot learning, where a model trained on a variety of tasks can be applied to entirely new ones without additional training. However, it's important to note that GRNPT likely learns some generalizable patterns from the training data, allowing it to perform well on unseen datasets related to the original training data. While it may not achieve true zero‐shot learning where no training is required, GRNPT's ability to transfer knowledge across different cell types and regulators represents a significant advancement in GRN inference.

## 4 Experimental Section

### Datasets

For benchmarking, we used ChIP‐seq data from 6 cell lines^[^ ^23^ ^]^ (**Table** **1**).

**Table 1:** The ChIP‐seq data used to train the model and validate.

Cell line	# of genes	# of TFs	# of interactions
Mouse Embryonic Stem Cells (mESC)	18 385	247	977 841
Human Embryonic Stem Cells (hESC)	17 735	130	436 563
Mouse Hematopoietic Stem Cells (mHSC) Erythroid lineage (mHSC(E)) Granulocyte‐Macrophage lineage (mHSC(GM)) Lymphoid lineage (mHSC(L))	4762	137	1 078 888
Mouse Dendritic Cells (mDCs)	7371	36	30 658

### Data Preprocessing

This study worked with raw count data containing over 4000 genes, and highly variable genes were selected using Scanpy. Scanpy is a widely used toolkit for single‐cell RNA‐seq analysis and is suited for handling large datasets due to its efficient data structures and scalable methods. This work used Scanpy for pre‐processing. Specifically, cells expressing fewer than 200 genes and genes present in fewer than three cells were removed. Cells with a high percentage of mitochondrial gene expression were also excluded based on dataset‐specific quantiles. Gene expression levels were normalized by scaling the total counts across all genes for each cell, followed by the identification of highly variable genes.

### Model Architecture

GRNPT was a GRN inference tool that can leverage public resources and learn from the training data (Figure 1A). To utilize public resources, gene embedding vectors generated by GenePT were incorporated into the model. Built on GPT‐3.5 frameworks using NCBI database, GenePT produces a 1536‐dimensional numerical vector for each gene.

### TCN Autoencoder

To Learn from scRNA‐seq Data, this work used a TCN Autoencoder. The Input data *X* ∈ *R* ^*C* × *L*^ is a tensor where *C* is the number of genes, and *L* is the number of cells. The encoder architecture comprises multiple TemporalBlock modules. Each TemporalBlock sequentially applies dilated convolutions, Chomp1d, ReLU activation, and Dropout. The encoding process can be formulated as follows:

*Y_i_* is the output of *i* th Temporal Block,

(1) $$Y_{i}=Dropout(ReLU(Chomp1d(Conv1d(X_{i}))))$$

*Y_i_* represents the transformed features after applying convolution, activation, padding removal, and dropout operations. *X_i_* is the input to the *i*‐th Temporal Block. Dropout is denoted as a regularization technique where randomly selected neurons are ignored during training, helping to prevent overfitting. ReLU is denoted as an activation function that outputs the input directly if it is positive, and outputs zero otherwise, introducing non‐linearity into the model and helping it learn complex patterns. Chomp1d is denoted as a function that removes extra padding introduced by dilated convolutions, ensuring the output length matches the original input size. Conv1d refers to a 1D convolutional operation that applies convolutional filters along the temporal or sequential dimension of the input data, capturing local dependencies.

After traversing N Temporal Block layers, the encoder generates a latent representation.

(2) $$Z=Encoder\left(X\right)$$

where $Z\in R^{C^{\prime }\times L}$ and *C*′ is the number of output channels of the last Temporal Block.

The decoder shares a similar architectural structure with the encoder, consisting of Temporal Block modules. It processes the latent representation *Z* to reconstructs the input $\hat{X}$,

For the *j*th TemporalBlock in the decoder,

(3) $${\hat{Y}}_{j}=\mathrm{Dropout}\left(\mathrm{ReLU}\left(\mathrm{Chomp}1d\left(\mathrm{Conv}1d\left(Z_{j}\right)\right)\right)\right)$$

The final output of the decoder is

(4) $$\hat{X}=\mathrm{Decoder}\left(Z\right)$$

where $\hat{X}\in R^{C^{\prime }\times L}$ matches the shape of the original input data.

Residual connections are incorporated into each Temporal Block to facilitate preservation of input information and enhance training stability.

(5) $$Y=\mathrm{ReLU}\left(Y_{2}+\mathrm{residual}\left(X\right)\right)$$

where *Y*_2_ is the output of the main branch of the current TemporalBlock, which represents the processed input after applying convolution, activation, and other transformations within that block. The residual denoted as residual (*X*) represents the input *X* potentially subjected to a downsampling operation to match output dimensions.

The mean squared error (MSE) loss function is employed to quantify the discrepancy between the original input and its reconstructed counterpart.

(5a) $$L\left(X,\hat{X}\right)=\frac{1}{C}\sum_{j=1}^{C}\sum_{K=1}^{L}{\left(X_{jk}-{\hat{X}}_{jk}\right)}^{2}$$

where X_jk_ refers to the value of the *j*‐th gene in the *k*‐th cell. ${\hat{X}}_{jk}$ refers to the reconstructed value corresponding to *X_jk_*. *C* is the number of genes in the dataset. *L* is the number of cells in the datasets.

### Transformer‐Based Link Prediction Model

To infer gene regulatory pairs, this work proposed a Transformer‐based link prediction model, which leverages the 2D gene features obtained from the LLM embedding and the TCN, respectively. These gene features, along with additional input features and a small set of labeled regulatory pairs (Figure 1C), are jointly used as inputs for the model.

The core components of this model included a multi‐head self‐attention mechanism and a feedforward neural network, designed for encoding and decoding node features. The model concatenated the features from LLM and the features TCN. When *C* is the number of cells, the input node features *x* ∈ *R* ^*C* × *F*^ represent gene features obtained through GenePT embedding, while the additional features *x_additional_*  ∈ *R* ^*C* × *F*′^represent temporal features derived from a TCN‐autoencoder. *F* corresponds to the number of features obtained from the GenePT embedding. *F*′ represents the dimensionality of the additional features. Initially, the model concatenates the input node features *x* with additional features *x_additional_*,

(6) $$h_{0}=\left[x,x_{\mathrm{additional}}\right]\in R^{C\times \left(F+F^{\prime }\right)}$$

The concatenated features are then processed through a linear layer to compute attention scores,

(7) $$a=W_{a}\;h_{0}+b_{a}\in R^{C\times 1}$$

where *W_a_* is the weight matrix of the linear layer, responsible for transforming the input features *h*_0_ into attention scores. *h*_0_ is the concatenated node feature matrix, which includes the gene expression features *x* and the temporal features *x*_additional_. *b_a_* is the bias term in the linear layer, which adjusts the computed attention scores.

The normalized attention weights (α) can be computed by applying the softmax function to the attention scores *a*.

(8) $$\alpha =\mathrm{softmax}\left(a\right)$$

The softmax function ensures that the attention weights sum to 1, allowing them to act as a weighted combination of the features.

These weights are applied to the concatenated features,

(9) $$h_{0}^{\mathrm{att}}=\alpha ⊙h_{0}$$

where ⊙ denotes element‐wise multiplication between the attention weights α and the concatenated feature matrix *h*_0_.

The attention‐weighted features are then linearly transformed to the hidden dimension.

(10) $$h_{1}=W_{e}\;h_{0}^{\mathrm{att}}+b_{e}\in R^{C\times H}$$

where *W_e_* is the weight matrix of the linear layer, which transforms the attention‐weighted features $h_{0}^{\mathrm{att}}$ into a higher‐dimensional hidden space. The matrix projects the features to a hidden dimension *H*. *b_e_* is the bias term added after the linear transformation to adjust the output features.

Next, the features are input into the multi‐head self‐attention layer,

(11) $$h_{2},\;\mathrm{attn}\_\mathrm{weights}\;=\mathrm{Multihead}\;\mathrm{Attention}\left(h_{1},h_{1},h_{1}\right)$$

where *h*_1_ serves as the query, key, and value. The output is *h*_2_, which represents the updated feature representation after attention is applied, and attn_weights, which contains the attention weights used to compute the output. MultiheadAttention splits the input features into multiple heads (subspaces), applies self‐attention independently to each head, and then concatenates the results.

This mechanism allows the model to capture dependencies between nodes on a global scale. The process is followed by layer normalization and a feedforward neural network,

(12) $$h_{3}=\mathrm{Layer}\;\mathrm{Norm}\left(h_{1}+\mathrm{Dropout}\left(h_{2}\right)\right)$$

where *h*_3_ represents the intermediate node features after applying layer normalization and dropout. Specifically, it is computed by adding the initial node embeddings *h*_1_ and the output from the multi‐head attention layer *h*_2_, followed by dropout and layer normalization. This step helps refine the node features before further processing. LayerNorm is a normalization technique that normalizes the input across the feature dimension.

The output of the feed forward neural network is

(13) $$h_{4}=\mathrm{Feed}\;\mathrm{Forward}\left(h_{3}\right)$$

Feed Forward is a standard fully connected layer (or set of layers) that processes the input features and applies non‐linear transformations.

(14) $$h_{5}=\mathrm{Layer}\;\mathrm{Norm}\;\;\left(h_{3}+\mathrm{Dropout}\left(h_{4}\right)\right)$$

The encoded node features *h*_5_ are then utilized in the decoding phase to predict the presence of links between node pairs. Specifically, for a given edge (*i*, *j*) with feature vectors *h_i_* and *h_j_*, the model predicts the link score as,

(15) $${\hat{y}}_{ij}=W_{f}\;\left[h_{i},h_{j}\right]+b_{f}$$

where *W_f_* is the weight matrix applied in the decoding phase. [*h_i_*,*h_j_*] denotes the concatenation of the feature vectors of nodes *i* and *j*. *b_f_* is the bias term added after applying the weight matrix *W_f_*.

The model is trained using a binary cross‐entropy loss function, which measures the discrepancy between the predicted link scores ${\hat{y}}_{ij}$ and the actual labels *y_ij_*. The loss function L can be

(16) $$L=-\frac{1}{M}\sum_{\left(i,j\right)\in E}\left(y_{ij}\log\left({\hat{y}}_{ij}\right)+\left(1-y_{ij}\right)\log\left(1-{\hat{y}}_{ij}\right)\right)$$

where *M* is the total number of labeled edges in the set. *E* denotes the set of labeled edges, *y_ij_* is the ground truth label (1 for a positive link, 0 for a negative link), and ${\hat{y}}_{ij}$ is the predicted probability of a link.

### Calculation of AUROC and AUPRC

AUROC was a widely used metric for evaluating the performance of a classifier.^[^ ^45^ ^]^ The receiver operating characteristic (ROC) curve illustrated the trade‐off between the true positive rate (TPR) and the false positive rate (FPR) at various threshold settings. AUROC was the area under ROC curve. After obtaining number of true positive (TP), false positive (FP), true negative (TN) and false negative (FN),^[^ ^46^ ^]^ FPR and TPR can be defined as

(17) $$\mathrm{FPR}\;=\mathrm{FP}/\left(\mathrm{FP}+\mathrm{TN}\right)$$

(18) $$\mathrm{TPR}=\mathrm{TP}/\left(\mathrm{TP}+\mathrm{FN}\right)$$

AUPRC is another metric for evaluating classifier performance, particularly for imbalanced datasets.^[^ ^47^ ^]^ The precision‐recall curve illustrates the relationship between precision and recall at various threshold settings.

(19) $$\left(\mathrm{Precision}=\mathrm{TP}\right)/\left(\mathrm{TP}+\mathrm{FP}\right)$$

Recall is the same with TPR. AUPRC is the area under the precision‐recall curve.

## Conflict of Interest

The authors declare no conflict of interest.

## Author Contributions

K.J.W. designed the study and developed the idea and framework. G.Z.W. completed the model construction and wrote the paper. P.M. and H.K. helped design the experiments. All authors read and approved the final version of the manuscript.

## Supporting information



Supporting Information

## Data Availability

Data sharing is not applicable to this article as no new data were created or analyzed in this study.
