# Supplementary material for: Integrating Prior Knowledge Using Transformer for Gene Regulatory Network Inference
Source: Adv Sci (Weinh). 2024 Nov 28;12(3):2409990. doi: 10.1002/advs.202409990 (PMC11744656; doi:10.1002/advs.202409990)
Supplement: Supplementary file 1 — Supporting Information [file ADVS-12-2409990-s001.docx]

**Figure S1: Overall performance comparison for GRN predictors using average ROC curve.** Average ROC curves are calculated by averaging the predicted scores over 10 runs for each model.

**Figure S2: Performance comparison of GRNPT with competing methods across six datasets.** GRNPT, which utilized only 10% of the data for training, demonstrates superior performance over other supervised methods (GMFGRN, DGRNS, DEEPRIG) that used over 50% of the data for training. Across all datasets and evaluation metrics, GRNPT consistently outperforms both supervised and unsupervised methods, highlighting its effectiveness and efficiency in gene regulatory network inference.

**Figure S3: Performance Comparison of GRNPT with supervised GRN inference methods using 30% training data.** GRNPT and five other supervised methods, including TDL, dynDeepDRIM, and scTGRN, were evaluated across multiple datasets with 30% of the data used for training. GRNPT consistently demonstrates superior performance across all metrics.

**
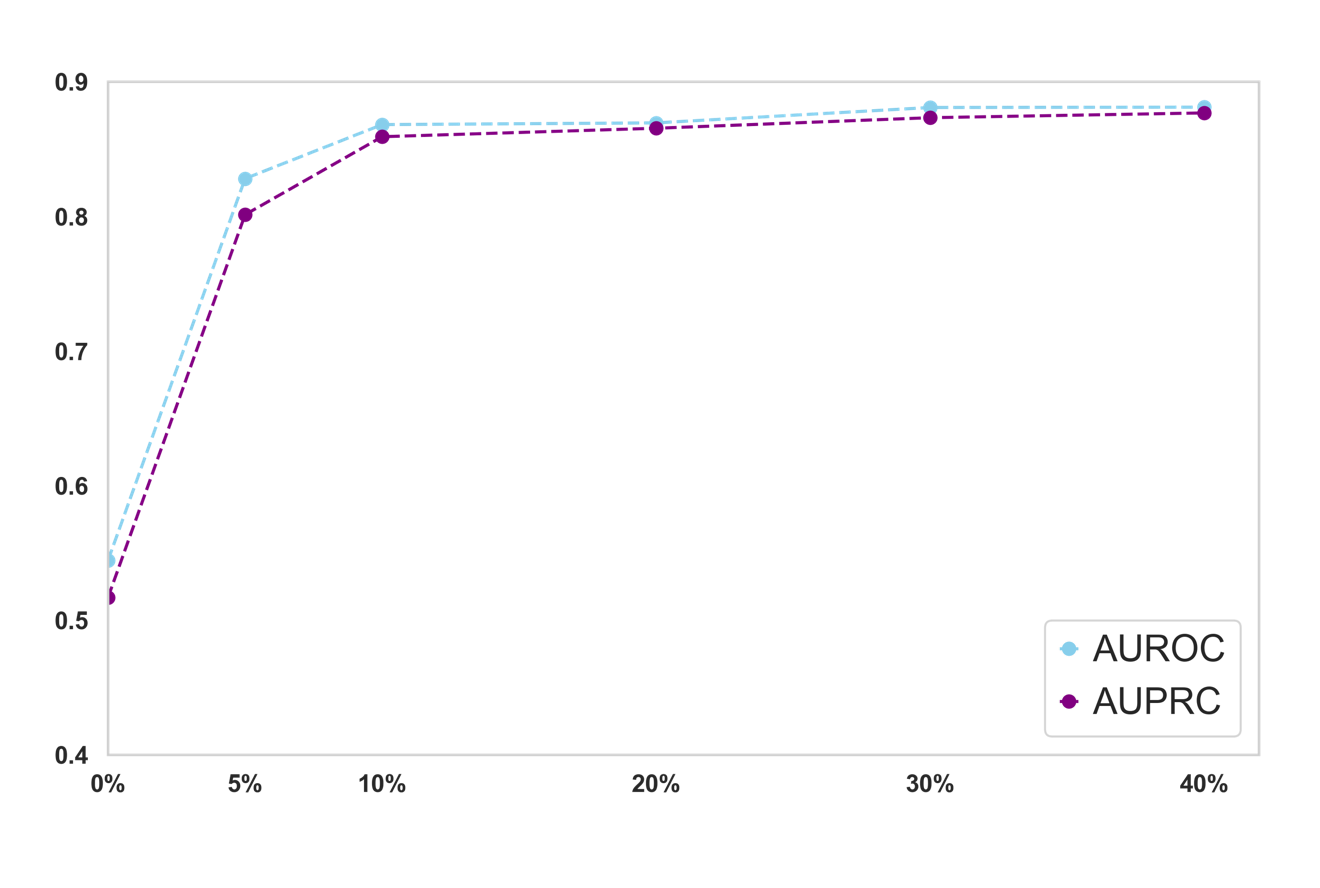
**

**Figure S4: Investigation on the training data size for GRNPT on mESC Dataset.**  GRNPT's performance on the mESC dataset was assessed using varying training data size. With less than 0.01% of the data, the model's accuracy was comparable to random guessing (AUROC and AUPRC ≈ 0.5). Increasing the training data to 5% significantly boosted performance to AUROC and AUPRC values around 0.8. The performance continued to improve with 10% of the training data with gradual but marginal gains for larger training datasets.

**Figure S5: GRNPT exhibits generalizability.** (A) GRNPT trained on two-thirds of mESC and hESC Data. (B) Investigation on the influence of the training data size for unseen datasets. GRNPT is trained for the mESC dataset and tested the mDC dataset.

**Figure S6: GRNPT is a robust approach for GRN inference** (A) GRNPT's performance on the mHSC-E dataset with varying cell counts (100-1000). (B) Performance under different dropout rates (10-90%). (C) Performance with varying numbers of genes (500-2000).
